# Marginal and internal fit of endo-crowns: AI-based evaluation

**DOI:** 10.6026/973206300212533

**Published:** 2025-08-31

**Authors:** Mahesh Suganna Golgeri, Gurpreet Singh, Pooja Arora, Urja Ahuja, Arun Kharavela Mohanty, Abhinav Patel

**Affiliations:** 1 Department of Prosthodontics, Riyadh ELM University, Riyadh, Kingdom Saudi Arabia; 2 Private practitioner, GDC Patiala, Punjab, India; 3 Department of Periodontology, Adesh Institute of Dental Science and Research, Bathinda, Punjab, India; 4 Department of Conservative Dentistry and Endodontics, Uttaranchal Medical and Dental Research Institute, Dehradun, Uttarakhand, India; 5 Department of Prosthodontics, Crown and Bridge, Kalinga Institute of Dental Sciences, Bhubaneswar, India; 6 Department of Public Health Dentistry, Rungta College of Dental Sciences and Research, Bhilai, Chhattisgarh, India

**Keywords:** Endo-crown, marginal adaptation, internal adaptation, impression techniques, digital impressions, conventional impressions, artificial intelligence (AI), Micro-CT CAD/CAM dentistry

## Abstract

The internal and marginal adaptation of endo-crown restorations fabricated using traditional and digital impression techniques is described. Artificial intelligence (AI) was used to analyze high-resolution imaging data obtained through digital scanning and micro-computed tomography. Results showed that endo-crowns produced with digital impressions exhibited superior fit compared to those made with conventional methods. AI-based analysis provided more accurate and consistent measurements than manual evaluation. Thus, we show AI's potential to enhance the precision of prosthodontic assessments.

## Background:

Due to significant loss of coronal tooth structure, endodontically treated teeth frequently have compromised structures, requiring restorations that provide both durability and retention. Because they rely on adhesive bonding and use the pulp chamber for macro mechanical retention, endo-crowns have become a conservative and successful restorative option, particularly for molars. This reduces the need for intra-radicular posts and excessive tooth preparation [1, 2]. Endo-crowns' internal and marginal adaptation is crucial to their success. Inadequate internal adaptation can jeopardise mechanical stability and retention, while poor marginal fit can result in microleakage, secondary caries and periodontal issues [3]. Therefore, using precise impression techniques is essential to guaranteeing a precise fit. Traditional impressions have long been the norm, usually made with polyvinyl siloxane (PVS) materials. However, because of their speed, patient comfort and potential for increased accuracy, digital impression techniques utilising intraoral scanners (IOS) and CAD/CAM technology are becoming more and more popular [4, 5]. Dental diagnostics and treatment planning now have more options thanks to recent developments in artificial intelligence (AI). AI systems are capable of accurately and reliably analysing imaging data, especially those that are based on machine learning and computer vision. Through automated image segmentation and gap quantification, artificial intelligence (AI) can reduce operator-dependent variability in prosthodontics by facilitating the assessment of internal and marginal gaps [6, 7]. Few studies have used AI to assess restorative fit despite these technological advancements, especially when comparing various impression methods for endo-crowns. Using AI-based analysis tools to improve the evaluation's objectivity and accuracy. Therefore, it is of interest to compare and evaluate the internal and marginal adaptation of endo-crown restorations made using digital and conventional impression techniques.

## Methodology:

Comparing the internal and marginal adaptation of endo-crown restorations made with traditional and digital impression techniques was the aim of this *in vitro* experimental investigation. Thirty freshly extracted human mandibular molars of comparable size were chosen and kept in a 0.1% thymol solution until they were needed. Caries-ridden, cracked, or restored teeth were not included. A low-speed diamond disc under water cooling was used to decoronate every tooth 2 mm above the cementoenamel junction. Using a diamond bur and a high-speed handpiece, standardised endo-crown preparations were carried out with a central retention cavity 3 mm deep into the pulp chamber and a flat butt-joint margin.

## Grouping and impression techniques:

The teeth were randomly divided into two groups (n = 15 per group):

[1] Group A (Conventional impressions): Impressions were made using polyvinyl siloxane (PVS) material in a two-step putty-wash technique. Models were poured using type IV dental stone and scanned with a desktop scanner.

[2] Group B (Digital impressions): Direct intraoral scans were obtained using a TRIOS 3 intraoral scanner (3Shape, Copenhagen, Denmark) and digital models were sent directly for CAD/CAM fabrication.

All endo-crowns were designed using CAD software and milled from lithium disilicate blocks (IPS e.max CAD) using a 5-axis milling machine. Crowns were crystallized, finished and polished according to the manufacturer's instructions. No cementation was performed to ensure accurate adaptation assessment. Each crown was seated on its respective tooth and scanned using micro-computed tomography (micro-CT) (resolution: 10 µm). Adaptation was evaluated in the marginal, axial and pulpal areas.AI-based image analysis software developed in Python using OpenCV and a convolutional neural network (CNN) model was used to automate gap detection and measurement. The algorithm was trained on manually segmented images and validated for accuracy. Measurements were recorded at standardized points in all three regions and average gap values were calculated for each sample. Data were analyzed using SPSS v26.0 (IBM, Armonk, NY). Normality was assessed with the Shapiro-Wilk test. Independent samples t-test was used to compare mean gap values between the two groups. Statistical significance was set at p < 0.05.

## Results:

Micro-CT analysis revealed differences in the adaptation quality between endo-crowns fabricated using conventional and digital impression techniques. The average gap measurements (mean ± standard deviation, in µm) in the marginal, axial and pulpal regions for each group are summarized in Table 1 (see PDF). Table 1 (see PDF) show Comparison of the internal and marginal adaptation of endo-crowns fabricated using conventional and digital impressions. Values represent mean gap dimensions in micro meters (µm). A statistically significant difference (p < 0.05) was observed in all regions. The digital impression group (Group B) consistently demonstrated significantly lower gap values across all measured regions compared to the conventional impression group (Group A), indicating superior internal and marginal adaptation. The Shapiro-Wilk test confirmed the normal distribution of data (p > 0.05 for all comparisons) and independent samples t-tests confirmed statistically significant differences between the two groups in each region examined (marginal, axial and pulpal; p < 0.05). The percentage improvement achieved using digital impressions was calculated. A consistent reduction in mean gap values was observed across all regions, as shown in Table 2 (see PDF). Descriptive statistics for total adaptation values across all regions are summarized in Table 3 (see PDF). Group B consistently showed lower maximum and average gap dimensions. Shapiro-Wilk tests were used to assess the normality of the data. All groups and regions met the assumption of normality (p > 0.05), as shown in Table 4 (see PDF).

## Discussion:

In contrast to endo-crowns made with traditional polyvinyl siloxane impressions, the results of this study showed that endo-crowns made with digital impression techniques exhibited noticeably better internal and marginal adaptation. This supports earlier findings that digital workflows produce restorations with improved fit accuracy because they capture surface details more accurately and reduce dimensional distortion [7]. The precision of marginal and internal fit is crucial for the clinical success of endo-crowns, as improper adaptation can lead to microleakage and restoration failure. Zortuk *et al.* (2012) emphasized that internal discrepancies can compromise retention and stress distribution, highlighting the need for accurate fabrication techniques in ceramic restorations [8]. McLean and von Fraunhofer (1971) established the gold standard for marginal gaps (<120 µm) in restorations, forming the basis for evaluating clinical acceptability. Their findings underscore the importance of precise fit to minimize cement dissolution and periodontal complications [9]. Ender and Mehl (2015) demonstrated that digital impressions and CAD/CAM systems significantly improve marginal fit consistency compared to conventional methods, which supports integrating AI and digital workflows in endo-crown fabrication [10]. Dudley and Farook (2025) highlighted that marginal gap values in endocrowns are strongly influenced by both the fabrication method and the material used, with CAD/CAM techniques generally producing more consistent fits. The review also emphasized that variation in measurement techniques significantly impacts reported gap values, underscoring the need for standardized evaluation protocols [11]. Contrepois *et al.* (2013), in their systematic review, identified variation in fit based on fabrication technique, restoration type, and measurement method. Their work validates the use of AI-enhanced evaluations as a more standardized and reproducible approach to assessing fit parameters [12]. This study has limitations even with the encouraging results. Because it is an *in vitro* study, it does not take intraoral access, patient movement, or salivary contamination into consideration. Additionally, no cementation was done, which could have an impact on marginal adaptation in clinical settings. To confirm these results in practical contexts, more *in-vivo* research is necessary.

## Conclusion:

When compared to traditional methods, digital impression techniques showed noticeably better internal and marginal adaptation for endo-crown restorations. According to these results, digital workflows could improve long-term clinical results and restoration fit. It is advised that more *in vivo* research be done to validate these findings in clinical settings.
